# Nociceptor neurons promote IgE class switch in B cells

**DOI:** 10.1172/jci.insight.148510

**Published:** 2021-12-22

**Authors:** Shreya Mathur, Jo-Chiao Wang, Corey R. Seehus, Florence Poirier, Theo Crosson, Yu-Chen Hsieh, Benjamin Doyle, Seungkyu Lee, Clifford J. Woolf, Simmie L. Foster, Sebastien Talbot

**Affiliations:** 1 David Geffen School of Medicine, University of California, Los Angeles, Los Angeles, California, USA.; 2 F.M. Kirby Neurobiology Center, Boston Children’s Hospital, Boston, Massachusetts, USA.; 3 Department of Neurobiology, Harvard Medical School, Boston, Massachusetts, USA.; 4 Department of Pharmacology and Physiology, University of Montréal, Québec, Canada.; 5 Department of Molecular Biology, Massachusetts General Hospital, Boston, Massachusetts, USA.; 6 Department of Genetics, Harvard Medical School, Boston, Massachusetts, USA.; 7 Depression Clinical & Research Program, Massachusetts General Hospital, Boston, Massachusetts, USA.; 8 Department of Psychiatry, Harvard Medical School, Boston, Massachusetts, USA.

**Keywords:** Inflammation, Neuroscience, Allergy, Immunoglobulins, Pain

## Abstract

Nociceptors, the high-threshold primary sensory neurons that trigger pain, interact with immune cells in the periphery to modulate innate immune responses. Whether they also participate in adaptive and humoral immunity is, however, not known. In this study, we probed if nociceptors have a role in distinct airway and skin models of allergic inflammation. In both models, the genetic ablation and pharmacological silencing of nociceptors substantially reduced inflammatory cell infiltration to the affected tissue. Moreover, we also found a profound and specific deficit in IgE production in these models of allergic inflammation. Mechanistically, we discovered that the nociceptor-released neuropeptide substance P helped trigger the formation of antibody-secreting cells and their release of IgE. Our findings suggest that nociceptors, in addition to their contributions to innate immunity, play a key role in modulating the adaptive immune response, particularly B cell antibody class switching to IgE.

## Graphical abstract

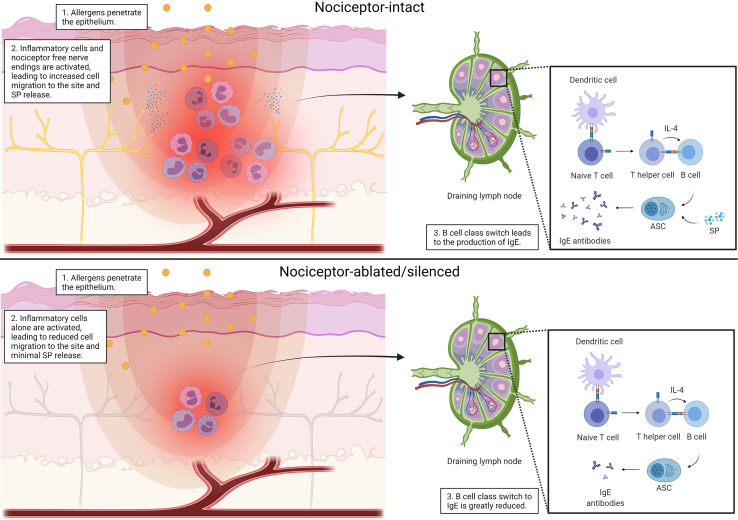

## Introduction

The coordinated interaction of the nervous and immune systems is critical for the body’s response to injury and infection. Local depolarization produced by noxious stimuli in the peripheral terminals of nociceptor sensory neurons initiates action potential firing, which triggers defensive reflexes and pain. There is also local neuropeptide release from the terminals as a consequence of the activation of voltage-dependent calcium channels (1, 2). This release of neuropeptides increases blood flow and vascular permeability, constituting neurogenic inflammation. There is growing evidence that in addition, sensory neurons also promote immune cell recruitment and dynamically regulate innate immune responses within the local tissue microenvironment (1, 3).

The crosstalk between sensory neurons and immune cells appears to be context dependent and varies depending on the sensory ganglia involved, the subset of neurons driving these effects, and the specific neuropeptides released. For example, somatosensory neurons inhibit T_H_1 and T_H_17 immunity in the context of bacterial (4) and fungal (5) infection. They do so mostly by secreting calcitonin gene–related peptide (CGRP), which modulates the activity of neutrophils (4) and CD301b^+^ dermal dendritic cells (5). On the other end of the spectrum, nociceptor neurons amplify T_H_2 immunity in allergic airway inflammation (6, 7), allergic dermatitis (8), and psoriasis (9). They do this by driving type 2 innate lymphoid cell (ILC2) (6, 7), CD4^+^ T cell (6), and dermal dendritic cell (9) activity. Optogenetic stimulation of nociceptors triggers rapid transcriptional changes in several leukocyte populations, including dendritic cells (3, 10). However, an effect of nociceptors on the B cell component of adaptive immunity, particularly antibody production, has not yet been identified.

B cells undergo class switch recombination, a process of DNA excision of immunoglobulin (Ig) heavy chain constant region genes, allowing a switch from IgM production to sequentially upstream IgD, IgG, IgE, and IgA production to mediate specific effector responses (11). IgE is a highly potent and tightly regulated antibody that is primarily responsible for allergic immune responses. In the setting of allergic disease, IgE-induced mast cell degranulation can rapidly lead to anaphylaxis and death (11, 12).

There is some evidence to support a crosstalk between immunoglobulins produced by B cells and nociceptors. High-affinity Fcγ receptor for IgG (FcγR1) and high-affinity IgE receptor (FcεR1) are expressed by sensory ganglion and myenteric plexus neurons (13), and IgE binds to FcεR1-expressing nociceptors (14), where they modulate pain hypersensitivity (15). Based on these findings, we recently discovered that lung-innervating jugular nodose complex ganglion neurons express FcεR1 and that the levels of this receptor increase in OVA-sensitized mice. We also showed that FcεR1γ-expressing vagal nociceptor neurons respond directly to OVA complexed with IgE, with both depolarization and neuropeptide release. The immunomodulatory neuropeptides directly amplify T_H_2 cell polarization while the specific deletion of FcεR1γ on nociceptors abolishes development of house dust mite–mediated allergic airway inflammation (16).

We aimed to address whether nociceptor neurons, in addition to amplifying innate type 2 allergic inflammation, also affect the production of IgE. We used either specific ablation of nociceptors in genetically engineered mice or the pharmacological silencing of the neurons using a charged sodium channel blocker to test if permanent loss or temporary silencing of nociceptors affects mucosal and skin immunity, with a focus on IgE class switch. Finally, we tested the immunomodulatory capacities of nociceptor-released neuropeptides on cultured B cell polarization and their release of antibodies.

## Results

### Allergic airway inflammation.

To investigate the role of nociceptors in allergic inflammatory responses, we used sensory neuron–ablated (TRPV1^cre/wt^ DTA^fl/wt^) mice engineered to express diphtheria toxin (DTA) in transient receptor potential cation channel subfamily V member 1–positive (TRPV1^+^) lineage cells, which leads to the neonatal elimination of most nociceptors (17). We used calcium microscopy to show an absence of capsaicin-responsive dorsal root ganglia neurons (Supplemental Figure 1, A–C; supplemental material available online with this article; https://doi.org/10.1172/jci.insight.148510DS1) and an increase in paw withdrawal latency in response to heat, to verify that the TRPV1^Cre/wt^ DTA^fl/wt^ mouse line showed a phenotypic reduction of thermonociception (Supplemental Figure 1D). Up to 95% of the TRPV1^Cre/wt^ DTA^fl/wt^ mice showed a reduction of nociception, and only these were selected for disease modeling. While TRPV1 presence on leukocytes has been suggested (18), it was not replicated by other investigators and has been refuted by several independent laboratories (2, 3, 16, 19–23). In addition to these functional data, unbiased single-cell and bulk (Immgen) RNA-sequencing data sets showed that *Trpv1* was not expressed by leukocytes, including T and B cell subpopulations (Supplemental Figure 1E) (16, 24).

**Figure 1:**
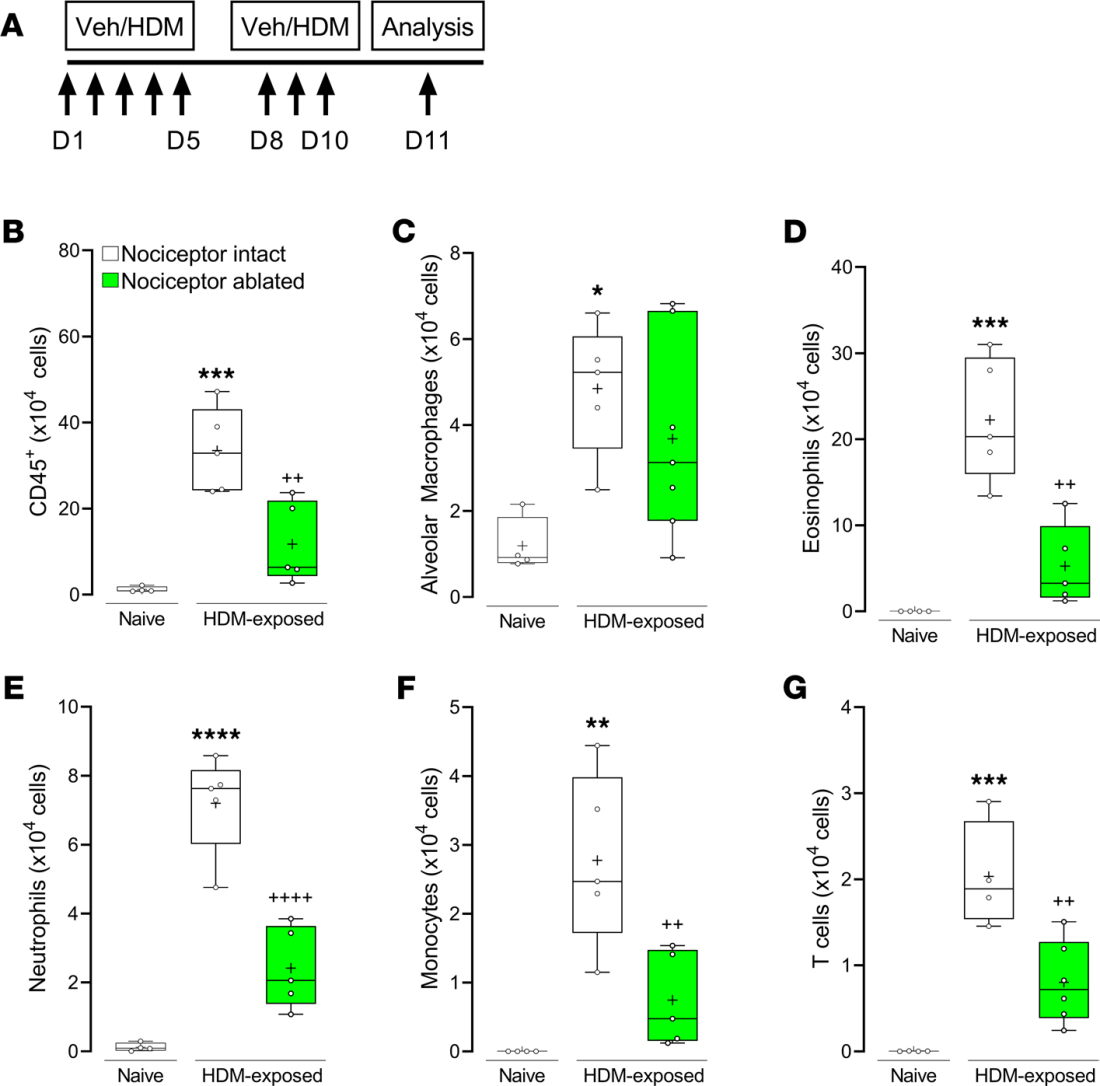
Nociceptor neurons contribute to allergic airway inflammation. (**A**) Allergic airway inflammation was induced by sensitizing littermate control (TRPV1^wt/wt^ DTA^fl/wt^) and genetically ablated (TRPV1^Cre/wt^ DTA^fl/wt^) mice to intranasal HDM (20 μg) daily on day 1 to day 5, followed by challenge to HDM from day 8 to day 10. Animals were sacrificed on day 11. Compared with vehicle-exposed mice, HDM challenge enhanced bronchoalveolar lavage fluid (BALF) numbers of (**B**) CD45^+^ cells, (**C**) alveolar macrophages, (**D**) eosinophils, (**E**) neutrophils, (**F**) monocytes, and (**G**) T cells. Genetic ablation of airway nociceptor neurons decreased HDM-mediated inflammation but retained recruitment of alveolar macrophages. Graphs show range, median, and “+” as mean. *P* values determined using 1-way ANOVA and Tukey’s multiple comparisons test. * denotes comparison with vehicle-exposed, nociceptor-intact mice and ^+^ comparison with HDM-exposed, nociceptor-intact mice. *P* < 0.05 is indicated by *; *P* < 0.01 is indicated by ** or ^++^; *P* < 0.001 is indicated by ***; *P* < 0.0001 is indicated by **** or ^++++^. Representative experiment is shown; *n* = 4–6/group. Experiments were replicated at least 3 independent times.

Using the TRPV1^Cre^ mouse line, we first assessed the impact of nociceptor neurons on an airway model of allergic inflammation induced by intranasal house dust mite (HDM) exposure (Figure 1A) (6). The HDM mouse model is well characterized as producing an eosinophilic inflammation with significant increases in IL-5 and IL-13, and these findings have been replicated in humans (25–27). We confirmed that HDM challenge enhanced bronchoalveolar lavage fluid (BALF) numbers of CD45^+^ cells (Figure 1B), alveolar macrophages (Figure 1C), eosinophils (Figure 1D), neutrophils (Figure 1E), monocytes (Figure 1F), and T cells (Figure 1G), as measured by flow cytometry. The genetic ablation (TRPV1^Cre/wt^ DTA^fl/wt^) of nociceptor neurons prevented the onset of HDM-mediated inflammation across all cell types identified in BALF, except for alveolar macrophages (Figure 1).

**Figure 2:**
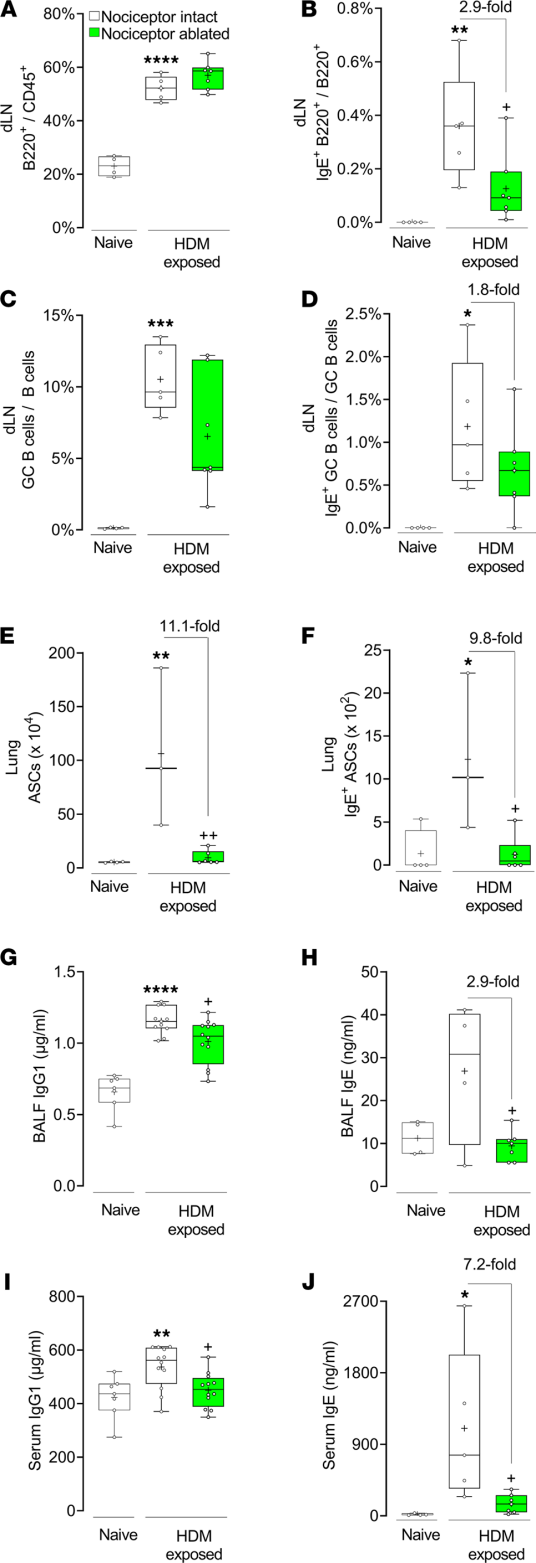
Nociceptor neurons are necessary for IgE production in allergic airway inflammation. HDM challenge increased the frequency of (**A**) total and (**B**) IgE^+^ B cells as well as (**C**) total and (**D**) IgE^+^ germinal center (GC) B cells in lung draining lymph nodes (dLNs). Nociceptor ablation significantly reduced the frequency of (**B**) IgE^+^ total B cells and (**D**) IgE^+^ GC B cells. HDM challenge increased the numbers of (**E**) total and (**F**) IgE^+^ antibody-secreting cells (ASCs) in the lungs of littermate control mice. Nociceptor ablation prevented this. HDM challenge increased IgG1 (**G** and **I**) and IgE (**H** and **J**) levels in bronchoalveolar lavage fluid (BALF) (**G** and **H**) and serum (**I** and **J**). These effects were absent in HDM-exposed TRPV1^Cre/wt^ DTA^fl/wt^ mice. Graphs show range, median, and “+” as mean. *P* values determined using 1-way ANOVA and Tukey’s multiple comparisons test. * denotes comparison with vehicle-exposed, nociceptor-intact mice and ^+^ comparison with HDM-exposed, nociceptor-intact mice. *P* < 0.05 is indicated by * or ^+^; *P* < 0.01 is indicated by ** or ^++^; *P* < 0.001 is indicated by ***; *P* < 0.0001 is indicated by ****. Representative experiment is shown; *n* = 4–7/group. Experiments were replicated at least 3 independent times.

We next phenotyped B cell responses in the lung and airway draining lymph nodes (dLNs). HDM challenge of naive mice increased the frequency of total (Figure 2A) and IgE^+^ B cells (Figure 2B and Supplemental Figure 2), total (Figure 2C) and IgE^+^ germinal center B cells (Figure 2D) in the dLNs, as well as total (Figure 2E) and IgE^+^ antibody-secreting cells (Figure 2F and Supplemental Figure 3) in the lungs. Then, we examined whether the increase in these B cell populations was reflected by changes in circulating antibodies and observed that BALF and serum levels of IgG1 (Figure 2, G and I) and IgE (Figure 2, H and J) were increased upon HDM challenge.

Along with the overall reduction in lung immune cell presence, we found that sensory neuron ablation reduced HDM-induced dLN IgE^+^ B cells (~3-fold; Figure 2B), lung IgE^+^ antibody-secreting cells (~10-fold; Figure 2F), BALF IgE levels (~3-fold; Figure 2H), and serum IgE levels (7.2-fold; Figure 2J). The proportion of IgE^+^ germinal center B cells (~1.8-fold decrease) in HDM-challenged, nociceptor-ablated mice was not significantly different from that of naive animals (*P* = 0.2; Figure 2D). While HDM challenge reduced the frequency of IgM^+^ germinal center B cells in the dLNs of both nociceptor-intact and -ablated mice to similar levels (Supplemental Figure 4, A and C), neither HDM challenge nor nociceptor ablation affected the frequency of total IgM^+^ B in the dLNs (Supplemental Figure 4B) or IgM^+^ antibody-secreting cells in the lungs (Supplemental Figure 4D). We next looked at the levels of antibody secretion by these cells and discovered that the absence of nociceptor neurons prevented HDM-mediated increase in BALF and serum levels of IgG1 (Figure 2, G and I) and IgE (Figure 2, H and J).

To confirm these findings, we used an extended version of the HDM protocol (Supplemental Figure 4E) devised to study the resolution of inflammation. Replicating the conclusions drawn from the data generated at the peak of inflammation (Figure 2), we found that nociceptor neuron ablation prevented the HDM-mediated increase in the number of BALF infiltrating leukocytes (Supplemental Figure 4F), BALF type 2 cytokine levels (Supplemental Figure 4G), and serum IgE levels (~4-fold; Supplemental Figure 4H).

### Atopic dermatitis.

**Figure 3:**
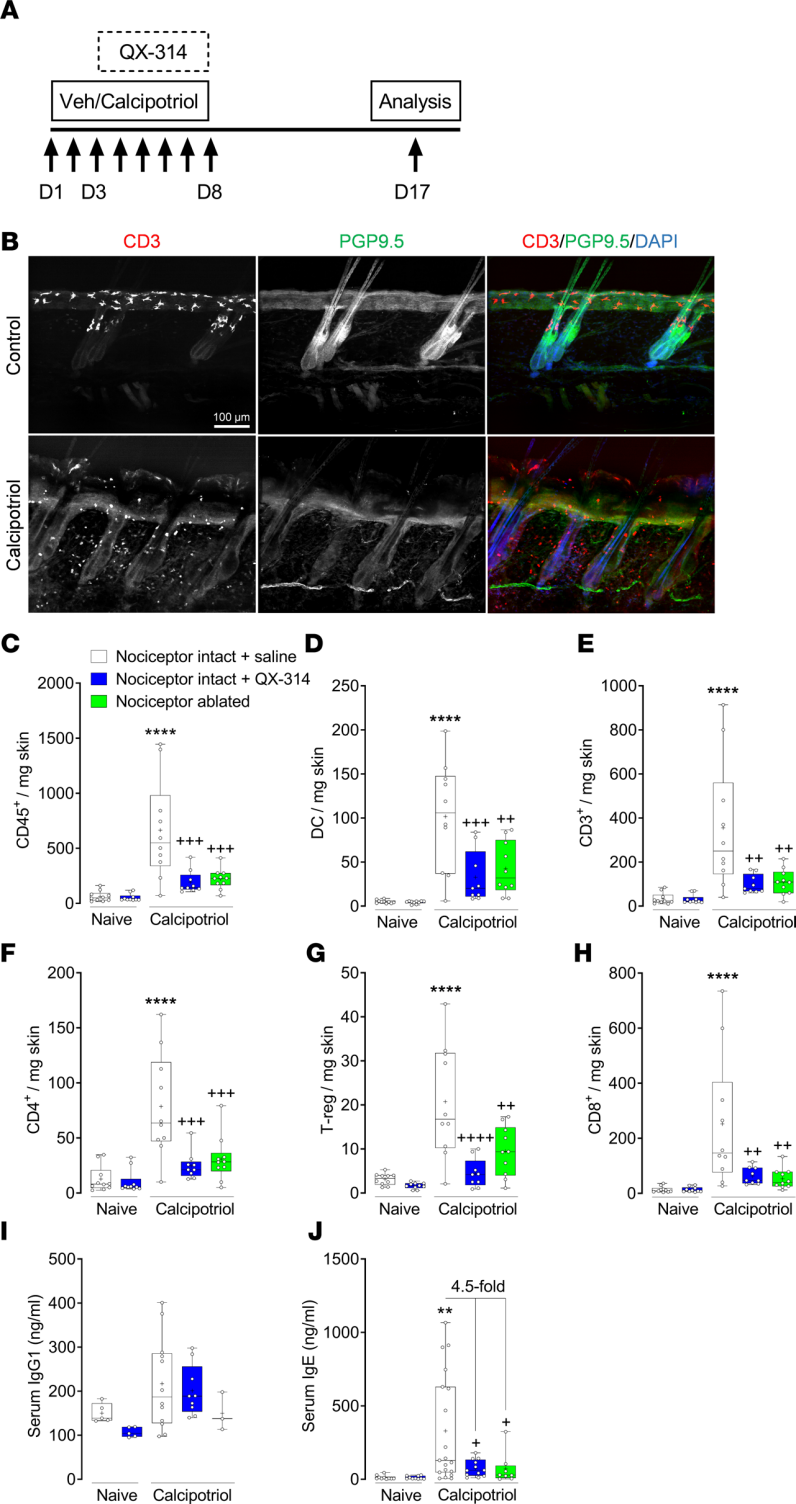
Nociceptor neurons affect allergic skin inflammation. (**A**) Timeline for induction of allergic skin inflammation using calcipotriol (vitamin D analog). Calcipotriol or vehicle (EtOH) was applied once daily on mouse dorsal skin for 8 days to littermate control or genetically ablated (TRPV1^cre/wt^ DTA^fl/wt^) mice. Groups of WT mice were additionally treated once daily with QX-314 (10 mg/mL, topical) from days 3 to 8. Animals were sacrificed on day 17. (**B**) Skin histology of day 5 control and calcipotriol-treated mice showed skin thickening, ECM deposits, loss of dendritic epidermal CD3^+^ T cells, recruitment of CD3^+^ T cells to inflamed tissue, and loss of PGP9.5 hair follicle innervation after sensitization. Scale bar represents 100 μm. Calcipotriol treatment increased numbers of (**C**) CD45^+^ cells, (**D**) dendritic cells, (**E**) CD3^+^ T cells, (**F**) CD4^+^ T cells, (**G**) regulatory T cells, and (**H**) CD8^+^ memory T cells in skin tissue. Nociceptor ablation (TRPV1^Cre/wt^ DTA^fl/wt^) reversed calcipotriol-mediated skin inflammation and significantly decreased (**J**) serum IgE levels. (**I**) Serum IgG1 levels were not affected by nociceptor ablation. Graphs show range, median, and “+” as mean. *P* values determined using 1-way ANOVA and Tukey’s multiple comparisons test. * denotes comparison with vehicle-exposed, nociceptor-intact mice and ^+^ comparison with calcipotriol-exposed, nociceptor-intact mice. *P* < 0.05 is indicated by ^+^; *P* < 0.01 is indicated by ** or ^++^; *P* < 0.001 is indicated by ^+++^; *P* < 0.0001 is indicated by **** or ^++++^. *n* = 3–5/group. Experiments were replicated at least 2 independent times.

After observing the impact of nociceptor ablation on lung immunity, we also evaluated the impact in a second inflammatory model with a different organ system because airway epithelial TRPV1 channels may play a role in the allergen response (22). For this, we used a classic model of atopic dermatitis induced by the daily topical application of calcipotriol (MC903), a vitamin D analog, on mouse dorsal skin (Figure 3A and Supplemental Figure 4I) (28, 29). In addition to this model of skin allergy, and given that the neonatal ablation of sensory neurons with TRPV1^cre/wt^ DTA^fl/wt^ mice could lead to compensatory changes, we also sought to verify our findings using a pharmacological silencing approach. To do so, we used a cationic derivative of lidocaine known as QX-314 to specifically silence nociceptor neurons. Briefly, QX-314 is a membrane-impermeant sodium channel blocker that enters open large-pore TRPV1 and transient receptor potential cation channel subfamily A member 1 channels on activated nociceptor terminals to block the channel on its cytoplasmic face and inhibit excitability (30, 31). Given that pruritogen-mediated entry of topical QX-314 has been demonstrated in the setting of allergic conjunctivitis (32), we allowed the natural course of inflammation to activate TRP channels on cutaneous nociceptor terminals and then evaluated the impact of QX-314–mediated nociceptor silencing.

Application of calcipotriol caused substantial erythema, pruritus, and xerosis of the affected skin. Skin histology revealed evidence of skin thickening, extracellular matrix deposition, loss of dendritic epidermal T cells, recruitment of T cells to inflamed tissue, loss of hair follicle innervation, and loss of tissue architecture after calcipotriol treatment (Figure 3B). Calcipotriol treatment also increased the number of CD45^+^ cells (Figure 3C), dermal dendritic cells (Figure 3D), CD3^+^ T cells (Figure 3E), CD4^+^ T cells (Figure 3F), regulatory T cells (Figure 3G), and memory CD8^+^ T cells (Figure 3H) in the skin, as measured by flow cytometry. Finally, the calcipotriol sensitization significantly increased serum levels of IgE (Figure 3J) but not IgG1 (Figure 3I).

As with the case of allergic airway inflammation, we found a significant reduction in immune cell recruitment to the skin across all cell types with sensory neuron ablation (TRPV1^Cre/wt^ DTA^fl/wt^) or by pharmacological silencing (QX-314; 10 mg/mL; Figure 3, C–J). Both nociceptor silencing and genetic ablation also decreased IgE levels but had no significant impact on IgG1 (Figure 3, I and J). As expected, in the absence of skin inflammation, QX-314 had no effect on any of the tested parameters (Figure 3, C–J). Neither calcipotriol sensitization nor sensory neuron modulation affected serum IgM levels (Supplemental Figure 4M).

Additionally, we examined B cell responses in the spleen. Calcipotriol sensitization did not significantly increase the frequency of splenic B cells (Supplemental Figure 4J) but raised the frequency of splenic germinal center B cells (~1.5-fold; Supplemental Figure 4K). Nociceptor silencing did not significantly affect these cell populations. Neither calcipotriol sensitization nor sensory neuron modulation affected the frequency of splenic IgM^+^ B cells (Supplemental Figure 4L). Changes in splenic IgE^+^ B cells could not be accurately assessed because of the inherently small splenic subpopulation (33).

### Type 1 inflammation.

An examination of type 1 skin inflammation induced by ovalbumin/complete Freund’s adjuvant (OVA/CFA) did not reveal a significant reduction in skin immunity (quantities of CD45^+^ cells, neutrophils, eosinophils, CD3^+^ T cells, CD8^+^ T cells, and regulatory T cells or neutrophil/eosinophil ratio) after nociceptor silencing or ablation (Supplemental Figure 5). As with our previous findings in the airways (2), these results indicate that nociceptor contribution to skin immunity also depends on the nature of the inflammation.

### Substance P drives B cell polarization.

**Figure 4:**
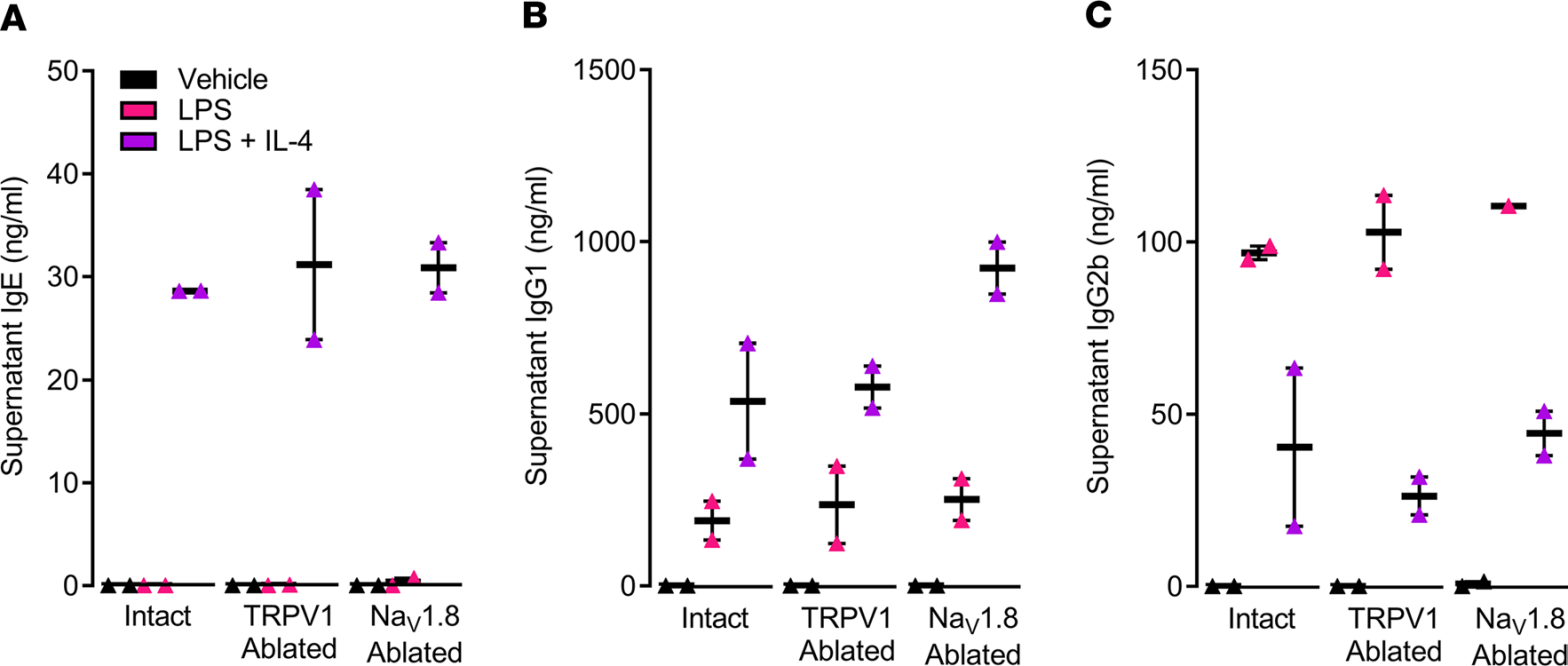
Neither TRPV1 nor Na_V_1. 8 neuron elimination reduces B cell ability to produce immunoglobulins. CD4^–^CD43^–^Ter-119^–^naive B cells were harvested from littermate control, TRPV1^Cre/wt^ DTA^fl/wt^ (labeled as TRPV1 ablated), and Na_V_1.8^Cre/wt^ DTA^fl/wt^ (labeled as Na_V_1.8 ablated) mouse spleens and cultured with/without LPS (25 μg/mL) and IL-4 (100 ng/mL). On day 5, culture supernatant was harvested and analyzed by ELISA. LPS (25 μg/mL) with/without IL-4 (100 ng/mL) increased B cells’ production of (**A**) IgE, (**B**) IgG1, and (**C**) IgG2b. These effects were not affected in any of the tested nociceptor ablation genotypes. Graphs show range and median. Representative experiment is shown. Experiments were replicated 2 independent times.

We examined whether the neonatal ablation of TRPV1^+^ (peptidergic nociceptors) or Na_V_1.8^+^ lineage (all nociceptors) sensory neurons triggers an intrinsic defect in B cell function during adulthood. To test this, naive splenic B cells (CD4^–^CD43^–^Ter-119^–^) were harvested from 8-week-old littermate control, TRPV1^cre/wt^ DTA^fl/wt^, and Na_V_1.8^cre/wt^ DTA^fl/wt^ mice. After 5 days of exposure to LPS (25 μg/mL) with/without IL-4 (100 ng/mL), B cells showed intact production of IgE (Figure 4A), IgG1 (Figure 4B), and IgG2b (Figure 4C), as measured in the cell culture supernatant by ELISA (34).

**Figure 5:**
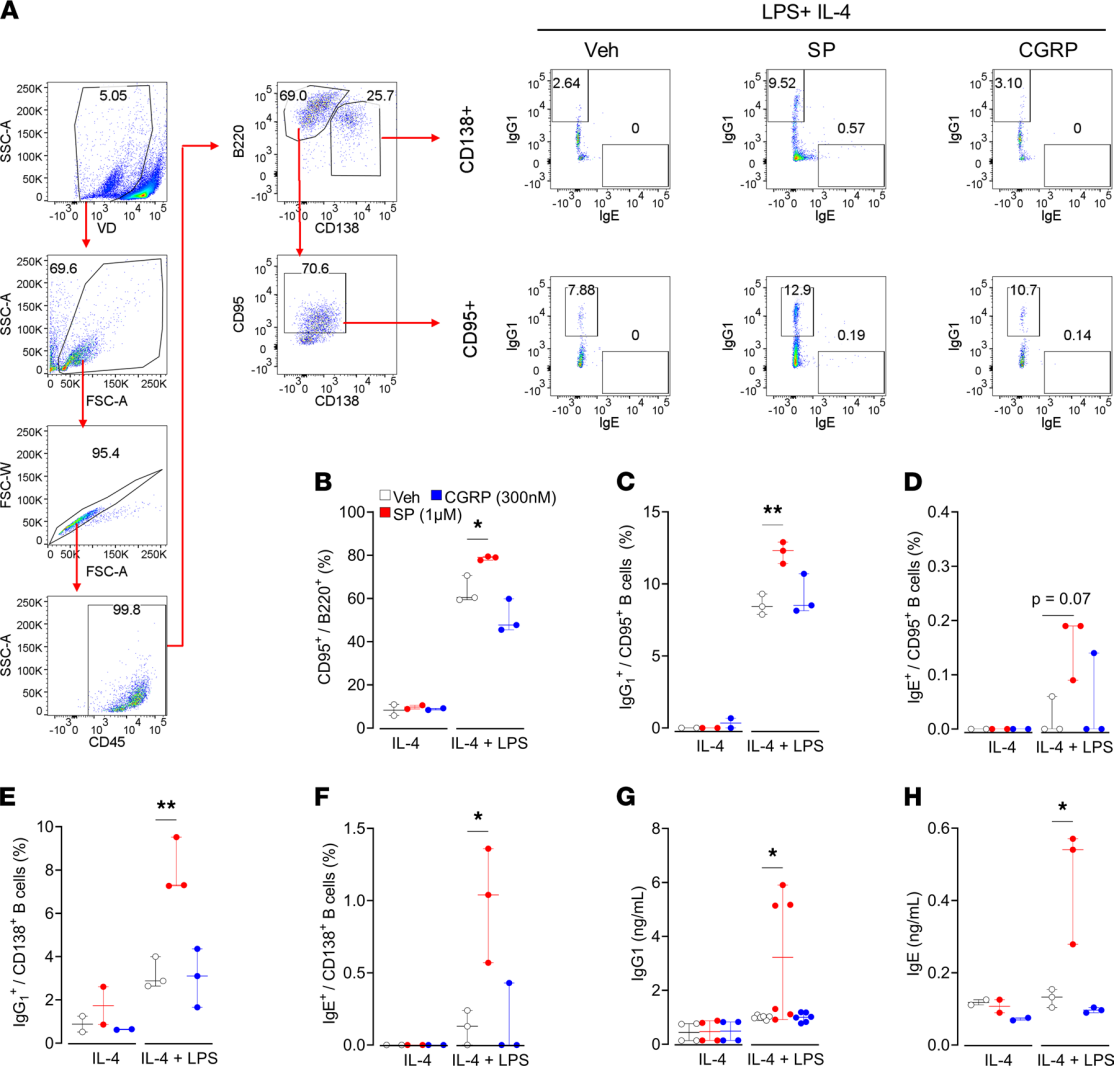
Substance P increases formation of antibody-secreting cells and their release of IgE. Splenic B cells were isolated from naive C57BL6 mice and cultured with or without LPS (10 μg/mL) and IL-4 (20 ng/mL) and exposed to vehicle, substance P (1 μM), and CGRP (300 nM). (**A**) After 4 days, the cells were immunophenotyped by flow cytometry, and immunoglobulin release was assessed by ELISA. LPS and IL-4 triggered the formation of (**B**) total, (**C** and **E**) IgG1^+^ and (**D** and **F**) IgE^+^ CD95^+^ (germinal center; **C** and **D**) and CD138^+^ (antibody-secreting cells; **E** and **F**) B cells. Additionally, LPS+IL-4 enhanced B cell release of (**G**) IgG1 and (**H**) IgE. These effects were augmented when B cells were coexposed to the neuropeptide substance P (1 μM). CGRP (300 nM) exposure had minimal impact on these parameters. Graphs show median and range. *P* values determined using 1-way ANOVA and Tukey’s multiple comparisons test. *P* < 0.05 is indicated by *; *P* < 0.01 is indicated by **. Representative experiment is shown; *n* = 3/group. Experiments were replicated at least 2 independent times.

Antibody production in naive B cells was not influenced by TRPV1^+^ or Na_V_1.8^+^ sensory neuronal ablation, which implies that the in vivo defect in IgE production after genetic ablation is not due to a developmental defect in B cell function but rather is a consequence of lack of modulation of class switch in these cells. In support of this hypothesis, B cell subtype transcriptome analysis revealed a basal expression of various neuropeptide receptors, including the CGRP receptors Ramp1 and Calcrl and the substance P receptors Tacr1, Mrgpra1, and Mrgprb2 (Supplemental Figure 1F). Based on these data, we exposed LPS+IL-4–stimulated B cells to substance P (1 μM) or CGRP (300 nM) and assessed their polarization and secretion of antibodies. Substance P enhanced formation of total (Figure 5, A and B), IgG1^+^ (Figure 5C), and IgE^+^ (Figure 5D) CD95^+^ (germinal center) B cells, as well as IgG1^+^ (Figure 5E) and IgE^+^ (Figure 5F) CD138^+^ (antibody-secreting cell) B cells (Figure 5, A–F, and Supplemental Figure 6). Additionally, substance P promoted B cell release of IgG1 (Figure 5G) and IgE (Figure 5H). CGRP (Figure 5, A–H, and Supplemental Figure 6) had no impact on the tested parameters. Overall, these data suggest that nociceptors, via the release of substance P, promote B cell polarization to germinal center B cells and antibody-secreting cells as well as the release of IgG1 and IgE. Because TRPV1^+^ sensory neurons express FcεR1 and respond to immune complexes by releasing substance P (13–16), we suggest that neurons and B cells partake in a feed-forward proinflammatory loop that amplifies adaptive immune responses.

## Discussion

Nociceptor neurons dampen innate immunity against pathogens and fungi (4, 5, 23, 35–39), but whether these neurons control humoral immunity remains unknown. Recent data support an upstream role for nociceptors in controlling adaptive immunity. For instance, nociceptors respond directly to the protease found in HDM (27), the immunoglobulins produced by B cells (16), as well as the proallergic cytokine IL-4 (40). In turn, these neurons locally release neuropeptides that promote conventional type 2 dendritic cell–mediated allergen trafficking to the lymph nodes (41), increase T_H_2 and ILC2 polarization (6, 7, 42), and induce mast cell degranulation (27). Substance P, one such neuropeptide, is increased in asthmatic patients’ lungs and promotes bronchial hyperresponsiveness (6), mucin imbalance (20, 43, 44), and coughing (45). Because these neurons detect immune complexes between IgE and allergens, and the genetic elimination of FcεR1 from TRPV1^+^ sensory neurons prevents the development of type 2 inflammation (13, 16, 46, 47), we postulate that the neurons contribute to humoral responses.

In support of this hypothesis, we discovered that the absence of TRPV1^+^ nociceptors by genetic ablation reduced serum IgE levels 7.2-fold in mice with allergic airway inflammation and about 4.5-fold in mice with dermatitis. The absence of these sensory neurons also reduced the number of dLN IgE^+^ B cells (~3-fold) and lung IgE^+^ antibody-secreting cells (~10-fold) without affecting the total number of dLN B cells and antibody-secreting cells. To put these data in perspective, the anti-IgE antibody omalizumab reduces circulating IgE by 5.5- to 7-fold in HDM-exposed mice (48). Dexamethasone and rapamycin lead to a 1.25- and 5-fold reduction, respectively (49, 50), while anti–inducible T cell costimulatory ligand therapy leads to a 5-fold reduction (51). Targeted immunotherapy with adjuvant poly-ɛ-caprolactone microparticles reduces IgE production in OVA-exposed mice 2-fold (52).

The decrease in circulatory IgE levels we observed cannot be attributed to an intrinsic impairment in B cell function in the mouse lines used to study these effects given that naive splenocyte-isolated B cells from TRPV1^cre/wt^ DTA^fl/wt^ and Na_V_1.8^cre/wt^ DTA^fl/wt^ mice readily class-switch in vitro when stimulated with classic signals (IL-4+LPS). In addition, while a few reports found *Trpv1* to be expressed by immune (e.g., CD4^+^ T cells; ref. 18) or stromal cells (53), the majority of investigators have found otherwise (2, 3, 16, 19–23), with at least 20 unbiased sequencing data sets (microarray, RNA sequencing, single-cell RNA sequencing) showing no significant *Trpv1* transcript expression by cell types other than neurons (10^7^-fold). Such an extensive absence of transcript expression across multiple studies renders TRPV1 expression on CD4^+^ T cells or airway epithelial cells less likely. Moreover, along with our genetic sensory neuronal ablation data, the local silencing of skin-innervating nociceptors, which requires the coexpression of TRP and Na_V_ channels in neurons, recapitulates the reduction in serum IgE levels. Altogether, our data highlight an important role for nociceptors in plasma cell IgE production.

TRPV1^+^ sensory neurons express FcεR1 and respond to IgE-allergen immune complexes by releasing substance P, which in turn amplifies T_H_2 cell production of IL-5 and IL-13 (13–16). Although at lower levels than in T_H_2 cells, B cell subtypes also express the substance P receptors Tacr1, Mrgpra1, and Mrgprb2. We show here that substance P, when coexposed with IL-4 and LPS, increases B220^+^ B cells’ polarization to CD95^+^ and CD138^+^ and increases their release of IgG1 and IgE. Taken together, these data provide a mechanistic link between nociceptor-produced substance P and IgE-producing plasma cells. These findings also highlight a potentially novel positive feedback loop that culminates in the amplification and maintenance of humoral immunity.

Consistent with an array of studies from late 1980s to early 1990s, our findings show that substance P enhances the late-stage activation and development of B cells as well as their immunoglobin synthesis in vivo and in vitro (54–57). Furthermore, our data support that this neuropeptide-enhanced humoral response also occurs in the allergic and IgE-mediated disease contexts. While the direct effects of neuropeptides on B cells and the expression of corresponding receptors have been demonstrated, recent findings show that the lymph node innervation by sensory neuron fibers is restricted to the superficial, macrophage-rich medulla and rarely found in the deep, lymphocyte-rich cortex (10, 58). Thus, how neuropeptides spatially reach B cells, infiltrating the lungs or inside the lymph node follicles to exert their effects remains to be tested.

Overall, our findings provide evidence for a substantial contribution of nociceptor neurons to humoral IgE responses. Given that topical silencing of nociceptors is sufficient to substantially reduce systemic IgE production, silencing these neurons may have therapeutic potential for allergic/IgE-mediated diseases, including food and skin allergies, rhinitis, and asthma. As a therapeutic, this may consequently reduce the risk of anaphylaxis and serious illness.

## Methods

Further information can be found in Supplemental Methods.

### Mice.

Mice were housed in standard environmental conditions (12-hour light/12-hour dark cycle; 23°C; food and water ad libitum) at facilities managed by Boston Children’s Hospital. C57BL/6J mice, 8 weeks old, were purchased from The Jackson Laboratory and were used as the WT condition in all experiments. TRPV1-Cre knockin mice (The Jackson Laboratory: 017769) were also purchased, and Na_v_1.8 CRE-RFP mice were supplied by Rohini Kuner (Heidelberg University, Heidelberg, Germany). Mouse lines with genetically ablated TRPV1 nociceptors (TRPV1^Cre/wt^ DTA^fl/wt^) and genetically ablated Na_v_1.8 nociceptors (Na_V_1.8^Cre/wt^ DTA^fl/wt^) as well as littermate controls for each (TRPV1^wt/wt^ DTA^fl/wt^; Na_V_1.8^wt/wt^ DTA^fl/wt^) were generated by crossing male heterozygous Cre mice to female homozygous *loxP* mice. Mice were used from ages 8 to 12 weeks. Offspring were tail clipped, and tissue was used to assess the presence of the transgene by standard PCR. Paw withdrawal latency to radiant heat stimulus using the Hargreaves test 1 day after OVA/CFA/incomplete Freund’s adjuvant s.c. injection (OVA 1 mg/mL in a 200 μL emulsion of sterile PBS and 50% CFA on day 0 and with 50% incomplete Freund’s adjuvant on day 7) was used to verify that the TRPV1^Cre/wt^ DTA^fl/wt^ and Na_V_1.8^Cre/wt^ DTA^fl/wt^ mouse line showed phenotypic reduction of nociception (2). Na_V_1.8^Cre/wt^ DTA^fl/wt^ mice were noted to have self-injurious behaviors (e.g., scratching to the point of bleeding), likely secondary to profound pain insensitivity, and were thus excluded from inflammatory models. Animals were excluded from the study if signs of physical injury or altercation were noted. Group randomization and blinding were not used in the experiments.

### HDM model.

HDM (CiteQ, 15J01) was resuspended in sterile saline (PBS) at a concentration of 400 μg/mL. Mice were lightly anesthetized (1.5%–2% isoflurane), and the suspension was administered intranasally, dropwise (20 μg/mouse in 50 μL). Mice were exposed daily to HDM on day 1 to day 5 and then challenged to HDM from day 8 to day 10. Nostrils used for administration were alternated each day. Mice were sacrificed either on day 11 (used to assess the peak of inflammation) or on day 15 (used to assess the speed of resolution of inflammation).

BALF, lung dLNs, lung tissue samples, and serum samples were collected for further analysis. For BALF collection, mice were anesthetized with urethane (200 μL i.p., 35%), and a 20 G sterile catheter was inserted longitudinally into the trachea. Then, 2 mL of ice-cold PBS containing protease inhibitors (Roche) was injected into the lung, which was then harvested and stored on ice. BALF underwent a 400*g* centrifugation (15 minutes; 4°C). The supernatant was harvested for ELISA and cells were resuspended in 200 μL. Blood was drawn from the heart for ELISA and allowed to coagulate at room temperature. Samples were centrifuged at 2000*g* for 20 minutes at 4°C and serum was harvested. If BALF was not collected, mice were euthanized with inhalation of carbon dioxide.

### Calcipotriol model.

Mice were lightly anesthetized (1.5% isoflurane in oxygen), and a small circular patch of their dorsal skin (radius ~5 mm) on the upper right corner of the back was carefully shaved. Mice that incurred cuts during the shaving process were not included. Calcipotriol (Tocris 112965-21-6) was resuspended in 100% ethanol (EtOH) as vehicle at a concentration of 4 nM and applied daily (10 μL/mouse) on the shaved patch of skin for 8 days. For mice in the QX-314 group, 10 mg/mL QX-314 (MilliporeSigma 112965-21-6) was added after calcipotriol addition from days 3 through 8. The mice were left untouched for an additional 8 days to allow for immune response. On day 17, mice were euthanized by inhalation of carbon dioxide, and blood was drawn from the heart for ELISA. Punch biopsy of the shaved skin was taken for flow cytometry.

### Immunohistochemistry.

Mice were perfused with 4% paraformaldehyde (PFA). Back hairy skin was shaved and dissected. Tissue was then processed with protocol adapted from Salz and Driskell (59). Skin was postfixed in 4% PFA for 15 minutes and washed twice in 1× PBS for 5 minutes each. Skin was trimmed, embedded in OCT, and frozen on dry ice. Once frozen, tissue was cryosectioned into 100 μm sections. Sections were stored in 1× PBS at 4°C. Sections were washed 3 times in 1× PBS, for 5 minutes each, and placed into a blocking solution of 1× PBS with 0.3% Triton X-100, 10% normal goat serum (NGS), and 3% bovine serum albumin for an hour. Sections were incubated in primary antibodies diluted in 1× PBS with 0.3% Triton X-100 and 2% NGS. Primary antibodies used were chicken anti-PGP9.5 (Abcam ab10404, 1:1000) and rabbit anti-CD3 (Abcam ab16669, 1:500). Tissue was incubated overnight on a seesaw rocker at room temperature. Sections were then washed in a well plate 3 times with 1× PBS, at 7 minutes each, before transfer into secondary antibody solution and incubation for 2 hours at room temperature. Secondary antibodies used were goat anti-chicken Alexa Fluor 488 (Invitrogen A11039, 1:1000) and goat anti-rabbit Alexa Fluor 647 (Invitrogen A21244, 1:1000). After secondary antibody incubation, sections were washed 3 times in 1× PBS, at 7 minutes each, and mounted with DAPI-containing mounting medium (Vector Laboratories). Mounted sections were imaged with a confocal microscope (Molecular Devices IXM-C) at 20× original magnification with numerical aperture of 0.45.

### B cell culture from genetically engineered mice.

Naive B cells (CD4^–^CD43^–^Ter-119^–^) were magnetically isolated from WT mouse spleens following the protocol of the MACS B Cell Isolation Kit (Miltenyi Biotec 130-090-862). B cell culture media were made using 430 mL RPMI 1640, 5 mL sodium pyruvate (Gibco: 11360-70), 5 mL 1 M HEPES buffer (Fluka 51558), 5 mL GlutaMAX (Thermo Fisher Scientific 35050061), 5 mL penicillin/streptomycin stock (Cellgro MT-3001-CI), 30 mL heat-inactivated fetal bovine serum stock (Invitrogen 10082-147), and 1.8 μL β-mercaptoethanol and vacuum filter sterilized (0.22 μm filter, MilliporeSigma SCGPU05RE). Isolated B cells were resuspended in these media (25°C) and diluted to a concentration of 1 × 10^6^ cells/mL. A total of 1 mL of cell solution was added to each well of a 6-well tissue culture plate. Stimulation cocktail was made at 2 times concentration using desired combinations of diluted LPS (stock 5 mg/mL) and IL-4 (stock 100 μg/mL). A total of 1 mL of the 2 times stimulation cocktail was added to each well of the plate, and cells were kept in a cell culture incubator (37°C, 5% CO_2_) for 4 days poststimulation. Supernatant was collected on day 4 poststimulation and frozen (–20°C) for subsequent ELISA.

### Neuropeptide stimulation of cultured B cells.

Splenic B cells were isolated from naive C57BL/6 mice by following the instructions of an EasySep Mouse B Cell Isolation Kit (STEMCELL Technologies 19854) and cultured at density of 2 × 10^6^ cells/well, 1 mL/well, in 24-well plates. Stimulation media were prepared by adding IL-4 (BioLegend 574306; 20 ng/mL) and LPS (MilliporeSigma L-4391; 10 μg/mL) in RPMI supplemented with FB Essence (Avantor Seradigm 10803-034; 10%), penicillin (100 IU/mL), streptomycin (100 μg/mL) (Corning 30-002-CI as mixed penicillin/streptomycin solution), and β-mercaptoethanol (Gibco 21985023; 55 μM). Neuropeptides αCGRP (Bachem AG 4025897.0500; 300 nM) or substance P (Tocris 1156; 1 μM) were added to the indicated groups. After 96-hour culture, supernatant samples were collected for ELISA, and cells were harvested for FACS analysis.

### Flow cytometry.

BALF cells were resuspended in FACS buffer and incubated with Fc block (0.5 mg/mL, 10 minutes; BD Biosciences). Cells were incubated on ice for 15 minutes, then centrifuged (500*g*, 5 minutes, 4°C), and the supernatant was decanted. BALF cells were stained with anti-CD45–BV421 (1:200; BioLegend catalog 103134), anti-Siglec-F–PE (1:200; Thermo Fisher Scientific catalog 12-1702-82), anti-CD11b-APC–Cy7 (1:200, BioLegend catalog 101262), anti-CD11c–FITC (1:200, BioLegend catalog 117306), anti-Ly-6G–APC (1:200, BioLegend catalog 127614), anti-Ly-6C–PE-Cy7 (1:200, BioLegend catalog 128018), and anti-CD90.2-PerCP–Cy5.5 (1:200, BioLegend catalog 140322) for 30 minutes in the dark at 4°C.

BALF singlet lymphocyte cell populations (used in Figure 1 and Supplemental Figure 2F) were defined as follows: alveolar macrophages Siglec-F^+^CD11c^+^; eosinophils Siglec-F^+^CD11c^–^; neutrophils Siglec-F^–^Ly-6G^+^Ly-6C^lo^CD11b^+^; monocytes Siglec-F^–^Ly-6G^–^Ly-6C^+^CD11b^+^CD11c^+^; T cells CD45^+^CD90.2^+^.

Lung and dLN single-cell suspensions were stained with Zombie Aqua (1:100 in PBS; BioLegend 423102) at room temperature in the dark for 15 minutes. Cells were washed with FACS buffer and incubated with normal rat serum (STEMCELL Technologies EasySep kit) at 4°C for 10 minutes to block nonspecific binding. Surface staining was then performed with anti-CD95–FITC (1:200; BioLegend catalog 152606), anti-B220–APC (1:200; BioLegend catalog 103212), anti-CD138–APC-Cy7 (1:100; BioLegend catalog 142530), and anti-GL7–PerCP-Cy5.5 (1:200; BioLegend catalog 144610) in the dark at 4°C for 20 minutes. Unconjugated anti-IgE (1:25; BioLegend catalog 406902) was used during surface staining to prevent nonspecific staining of surface-bound IgE. Cells were then fixed and permeabilized following the Fixation/Permeabilization Solution Kit manufacturer’s instructions (BD Biosciences 554714) and stained with anti-IgE–PE (1:50; BioLegend catalog 406908) and anti-IgM–PE (1:100; Thermo Fisher Scientific catalog 25-5790-82) in the dark at 4°C for 20 minutes. After staining, cells were washed twice with BD Biosciences Perm/Wash buffer.

Lung and dLN lymphocyte cell populations (used in Figure 2 and Supplemental Figure 2, B–D) were defined as follows: B cells CD45^+^B220^+^CD138^–^; germinal center B cells CD45^+^B220^+^CD138^–^GL7^+^CD95^+^; antibody-secreting cells CD45^+^B220^lo/–^CD138^+^.

For the skin inflammation model, the whole area of treated skin was harvested, cut into smaller sections, and washed with 500 μL liberase thermolysin low (TL) (MilliporeSigma) (1 mg/mL in double-distilled water). Skin samples were incubated in a solution of 0.5 mg/mL liberase TL (Roche 5401020001) and 0.5 mg/mL Hanks’ balanced salt solution (Thermo Fisher Scientific 14025076) in a bead bath at 37°C for 1 hour. Samples were transferred to 15 mL conicals containing 7 mL FACS buffer (15 mL heat-inactivated fetal bovine serum in 500 mL PBS) and filtered through a 70 μm mesh filter into 50 mL conicals. Then, 25 mL of PBS was added to each conical and centrifuged at 500*g* for 10 minutes at 4°C. Supernatant was aspirated and cells were resuspended in 5 mL FACS buffer. Then, 10 μL of the cell suspension was used for cell counts on a hemocytometer. A total of 500 μL of each cell suspension was transferred to 5 mL, round-bottom, polystyrene test tubes (Corning 352052) and incubated with Fc block (1:500, 45 minutes; BD Biosciences 553141) at 4°C. Samples were centrifuged (500*g*, 5 minutes, 4°C) and the supernatant was decanted. Cells from skin tissue samples were then stained for combinations of surface markers with anti–CD11c-FITC (BioLegend 117306), anti-Thy1.2–PacBlue (BioLegend 140306), anti-CD4–PerCP 710 (1:500; eBioscience 46-0041-82), anti-CD11b–APC (BioLegend 101212), anti-GR-1–APC (BioLegend 108412), anti-CD45–APC-Cy7 (1:500; BioLegend 103116), anti-CD3–PE (BioLegend 100206), and anti-Siglec-F–PE (BioLegend 155506), each at a 1:200 dilution unless otherwise noted, in FACS buffer for 30 minutes in the dark at 4°C. Cells were then washed with 3 mL FACS buffer, centrifuged (500*g*, 5 minutes, 4°C), and resuspended in 1 mL FACS buffer for further analysis.

Skin singlet lymphocyte cell populations (used in Figure 3 and Supplemental Figure 3) were defined as follows: CD8^+^ memory T cells Lin^–^ (CD19, CD11b, CD11c), CD45^+^CD3^+^CD4^–^; eosinophils CD45^+^CD11b^+^Siglec-F^+^Gr1^–^CD3^–^; neutrophils CD45^+^Gr1^+^CD11b^+^Siglec-F^–^CD3^–^; macrophages CD45^+^CD11b^+^Gr1^–^Siglec-F^–^CD3^–^; dendritic cells CD45^+^CD11b^+^CD11c^+^Gr1^–^Siglec-F^–^CD3^–^; T cells CD45^+^CD3^+^CD11b^–^Siglec-F^–^Gr1^–^; regulatory T cells CD45^+^CD3^+^CD25^+^Siglec-F^–^CD11b^–^Gr1^–^.

Spleen samples were mashed between glass slides and rinsed with double-distilled water (MilliporeSigma: Z00QSV0WW) into a single-cell suspension. The solution was allowed to sediment, and the cells in suspension were transferred for further use. The cells were centrifuged (500*g*, 5 minutes, 4°C) and the supernatant was aspirated. Red blood cell lysis buffer (1 mL, 1 minute) was added, and the reaction was stopped by flooding with FACS buffer. Cells were resuspended in 10 mL FACS buffer. Then, 10 μL of the cell suspension was used for cell counts on a hemocytometer. A total of 200 μL of each cell suspension was transferred to 5 mL, round-bottom, polystyrene test tubes and incubated with Fc block (1:500, 45 minutes) at 4°C. Samples were centrifuged (500*g*, 5 minutes, 4°C) and the supernatant was decanted. Cells from spleen tissue samples were stained for surface markers with anti-CD3–PacBlue (BioLegend 100214), anti-PD1–FITC (BioLegend 135214), anti-B220–PerCP 710 (eBioscience 46-0452-80), anti-B220–PE (BioLegend 103208), anti-Ki67–PE (BioLegend 652404), anti-Ki67–PerCP 710 (eBioscience 46-5698-82), and anti-GL7–APC (BioLegend 144602), each at a 1:200 dilution in FACS buffer for 30 minutes in the dark at 4°C. Cells were then fixed and permeabilized using a Fixation/Permeabilization Solution Kit following the manufacturer’s instructions (BD Biosciences 554714). Cells were then stained for intracellular markers using anti-IgA–APC-Cy7 (SouthernBiotech 0106-19), anti-IgD–APC-eFluor 780 (eBioscience 7-5993-80), and anti-IgM–APC-eFluor 780 (Thermo Fisher Scientific 47-5790-80), each at a 1:200 dilution in FACS buffer for 30 minutes in the dark at 4°C. After staining, cells were washed twice with BD Biosciences Perm/Wash buffer. IgE staining was performed using the methods described by Gallagher et al. with anti-IgE–FITC (1:100, BioLegend 406906) (60).

Spleen singlet lymphocyte cell populations (used in Supplemental Figure 2, J–L) were defined as follows for the calcipotriol model: B cells CD45^+^CD4^–^B220^+^; germinal center B cells CD45^+^CD4^–^B220^+^GL7^+^IgD^–^.

Data were acquired with a FACSCanto II (BD Biosciences). Total cell counts were performed using a standard hemocytometer, with absolute cell numbers calculated as total cell number multiplied by the percentage of the cell subpopulation as determined by flow cytometry. Data were analyzed using FlowJo v10 (BD Biosciences), and representative cell populating gating schemes are presented in Supplemental Figures 2 and 3.

### ELISA.

Antibody concentrations in collected blood and supernatant were determined using IgE (BioLegend 432401), IgM (Thermo Fisher Scientific 88-50470-22), IgG1 (Bethyl Laboratories E90-105), IgG2a (Bethyl Laboratories E99-107), and IgG2b (Bethyl Laboratories E99-109) ELISA kits. Freshly collected blood samples were prepped for ELISA by first allowing samples to clot and then centrifuging at 1500*g* for 15 minutes. The clear plasma, not including the buffy coat, was collected and frozen (–20°C) for subsequent ELISA. The serum samples were diluted at 1:50 for IgE ELISAs or 1:1000 for IgG1, IgG2a, IgG2b, and IgM ELISAs in the respective kit sample diluent. Supernatant collected from in vitro cultures was used undiluted for the IgE ELISA and diluted 1:5 for all other ELISAs. Differences in ELISA dilutions were equalized prior to analysis. Kit protocols were followed for standards and addition of capture antibody, detection antibody, TMB, and stop solution (2N sulfuric acid). For OVA-specific ELISAs, 96-well ELISA plates were instead coated with 1 μg/mL OVA (MilliporeSigma) and incubated overnight at 4°C, and then kit protocols were followed (61). Absorbance was measured at 450 nm wavelength.

### Single-cell RNA sequencing.

Using the publicly available Broad Institute single-cell portal, we performed an in silico analysis of single-cell RNA sequencing of mouse lung CD45^+^ immune cells and deposited the data in Gene Expression Omnibus (GEO accession: GSE127465).

### Statistics.

Details of individual experiments are provided in the figure legends. Effect size was calculated using pilot data comparing total serum IgE in 8 HDM mice (mean 529.66 ng/mL, SD = 152.69) and 5 HDM TRPV1^Cre/wt^ DTA^fl/wt^ mice (mean 5.05 ng/mL, SD = 2.79), treated as described above. Power analysis for testing the difference between 2 independent sample means showed that a sample size of 3 per group is required to detect differences between 2 means with a power of 0.995 or higher at the significance level of 0.05. All power analysis and sample size calculations were performed using the software G*Power 3.1.9.7. Data are expressed as mean ± SEM unless otherwise noted. Statistical significance was calculated using GraphPad Prism 8 software and determined by 1-way ANOVA and Tukey’s multiple comparisons test. *P* values less than 0.05 were considered significant. Outliers were determined as data points more than 1.5 IQRs below the first quartile or above the third quartile. Numbers of animals are defined in figure legends. All experiments were replicated.

### Study approval.

All procedures were approved by the Institutional Animal Care and Use Committee of Boston Children’s Hospital.

## Author contributions

SM, JCW, CRS, ST, SLF, and CJW designed the study. SM, CRS, JCW, FP, TC, YCH, BD, SL, and SLF conducted the experiments. SM, CRS, JCW, YCH, BD, SL, ST, and SLF analyzed the data. SM, ST, SLF, and CJW wrote the manuscript. SM and SLF initiated the study, so SM is listed before JCW in the author list.

## Supplementary Material

Supplemental data

## Version 1 11/02/2021

In-Press Preview

## Version 2 12/22/2021

Electronic publication
