# Supplementary material for: Nociceptor neurons promote IgE class switch in B cells
Source: JCI Insight. 2021 Dec 22;6(24):e148510. doi: 10.1172/jci.insight.148510 (PMC8783686; doi:10.1172/jci.insight.148510)
Supplement: Supplemental data [file jciinsight-6-148510-s206.pdf]

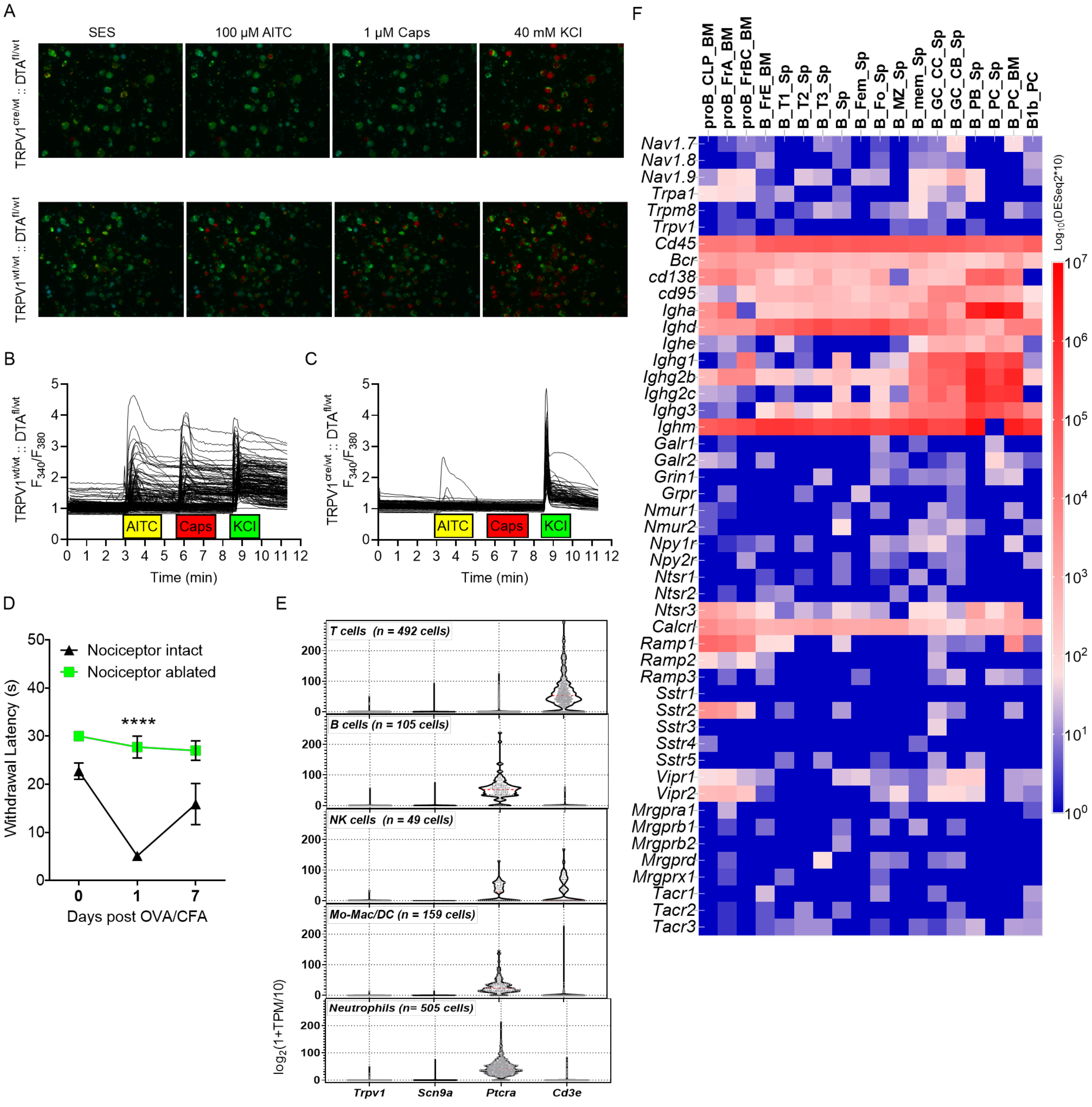

Supplemental Figure 1. Genetic ablation of TRPV1 neurons results in hyposensitivity to capsaicin and heat. (**A-C**) AITC (100μM; 30 seconds) and capsaicin (1μM; 30 seconds) induce calcium flux in naive mouse cultured (for 24 hours) DRG neurons, but not in TRPV1$^{Cre/wt}$::DTA$^{fl/wt}$ mouse neurons. KCl (40mM; 10 seconds)-induced calcium flux is observed in neurons from both mouse lines.

(**D**) One day after the injection of CFA/OVA, wildtype mice present with a transient thermal hypersensitivity which self-resolve after 7 days. The thermal hyperalgesia is absent in sensory neuron depleted (TRPV1$^{Cre/wt}$::DTA$^{fl/wt}$) mice.

(**E**) In-silico analysis of single-cell RNA-sequencing of mouse lung immune cells revealed high transcript levels of and *CD3e*, but no expression of TRPV1 or Nav1.8. *Individual cell data are shown as a log₂ of 1 + (transcript per million/10). The dashed red line represents the median. Experimental details and cell clustering were defined in Zilionis et al., 2019.*

(**F**) RNA sequencing of B cell subpopulations revealed their basal expression of *Cd45* and *B220*. They also express various neuropeptide receptors including the CGRP receptors *Ramp1* and *Calcrl*. In contrast, B cell subpopulations do not express *Trpv1*. *Heatmaps data are shown as DESeq2 on a logarithmic scale. Experimental details and cell clustering are available via the Immgen database.*

*****P<0.0001 by two-way ANOVA and Sidak's multiple comparisons test. Representative experiment is shown; n=8-492/group. Experiments were replicated at least two independent times.*

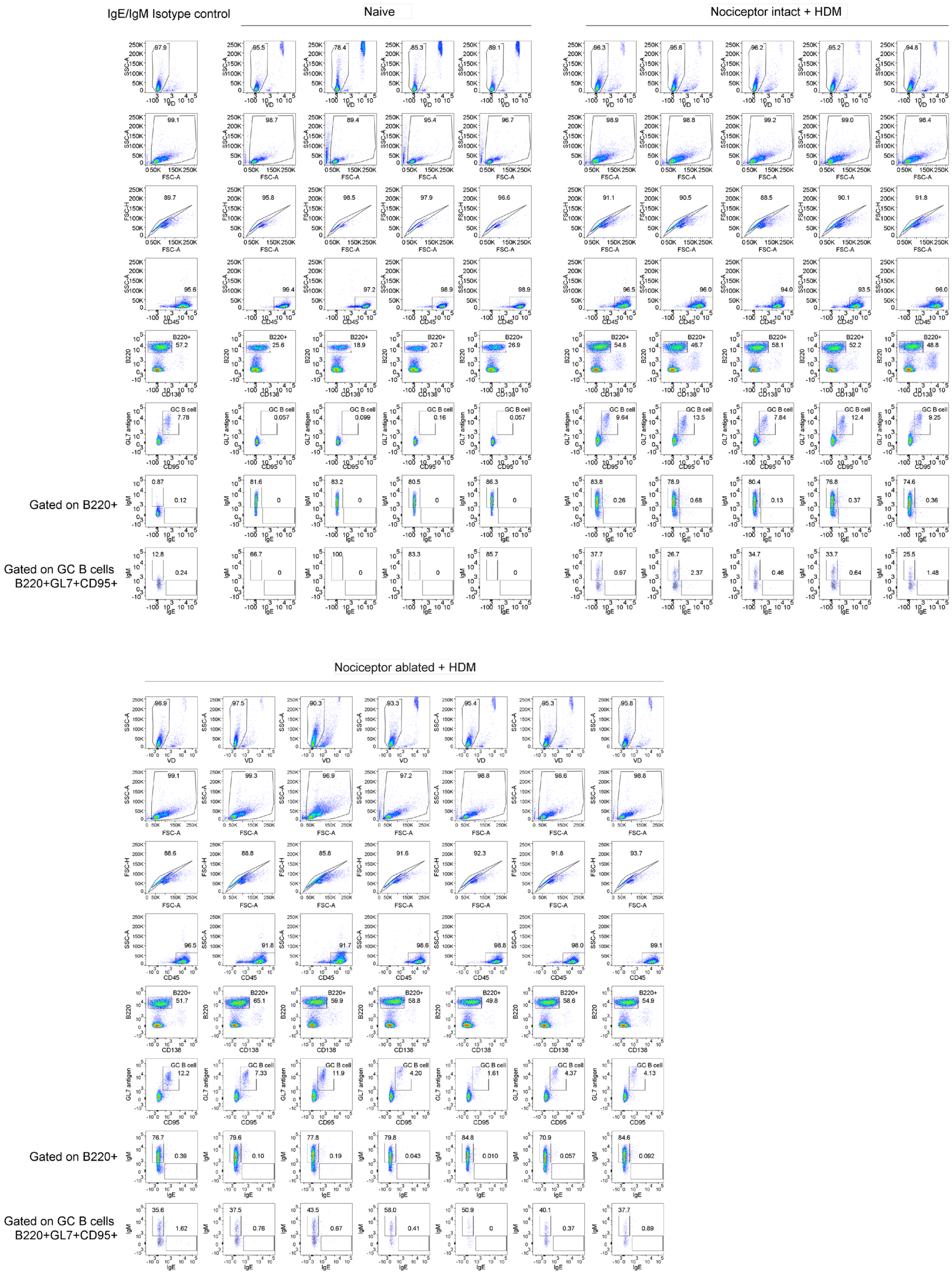
IgE/IgM Isotype control
Naive
Nociceptor intact + HDM
Gated on B220+
Gated on GC B cells
B220+GL7+CD95+
Nociceptor ablated + HDM
Gated on B220+
Gated on GC B cells
B220+GL7+CD95+

Supplemental Figure 2. Draining lymph node immunophenotyping strategy**.** Allergic airway inflammation was induced by sensitizing nociceptor-intact ($TRPV1^{wt/wt}$::$DTA^{fl/wt}$) or nociceptor-ablated ($TRPV1^{Cre/wt}$::$DTA^{fl/wt}$) mice to intranasal HDM (20µg) daily on day 1 to day 5, followed by challenges to HDM from day 8 to day 10. Animals were sacrificed on day 11. Lung draining lymph nodes were harvested and immunophenotyped by flow cytometry. Data were compared with draining lymph nodes harvested from naïve mice. Shown are the representative gating strategy for draining lymph node $B220^+$ cells and germinal center B cells (defined as $CD45^+B220^+CD138^-GL7^+CD95^+$). IgE isotype control antibody staining is shown for comparison.

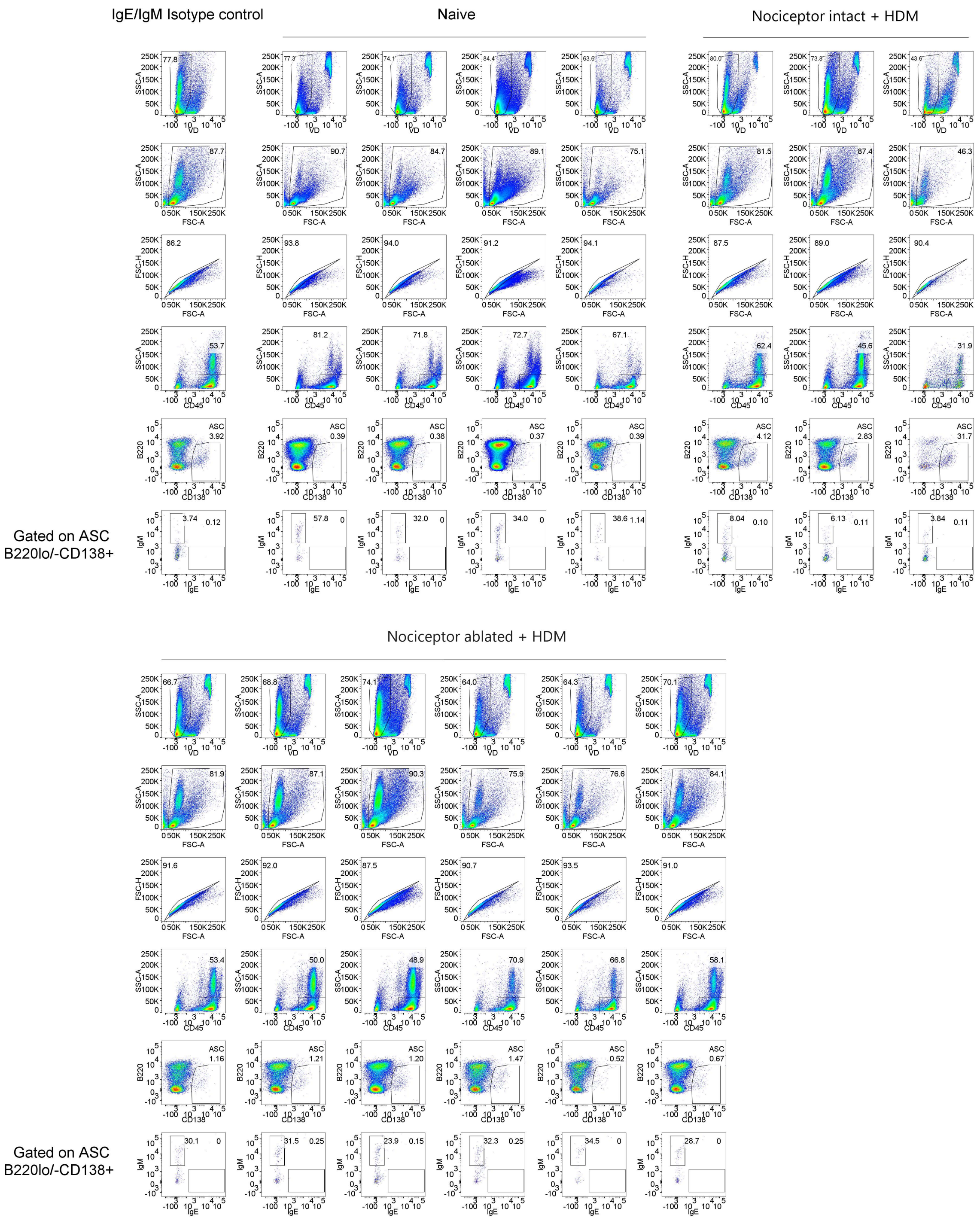

Supplemental Figure 3. Lung immunophenotyping strategy. Allergic airway inflammation was induced by sensitizing nociceptor-intact (TRPV1$^{wt/wt}$::DTA$^{fl/wt}$) or nociceptor-ablated (TRPV1$^{Cre/wt}$::DTA$^{fl/wt}$) mice to intranasal HDM (20μg) daily on day 1 to day 5, followed by challenges to HDM from day 8 to day 10. Animals were sacrificed on day 11. BALFs were harvested and immunophenotyped by flow cytometry. Data were compared with BALF harvested from naïve mice. Shown are the representative gating strategy for lung antibody secreting cells (defined as CD45^{+}B220$^{lo/-}$CD138^{+}). IgE isotype control antibody staining is shown for comparison.

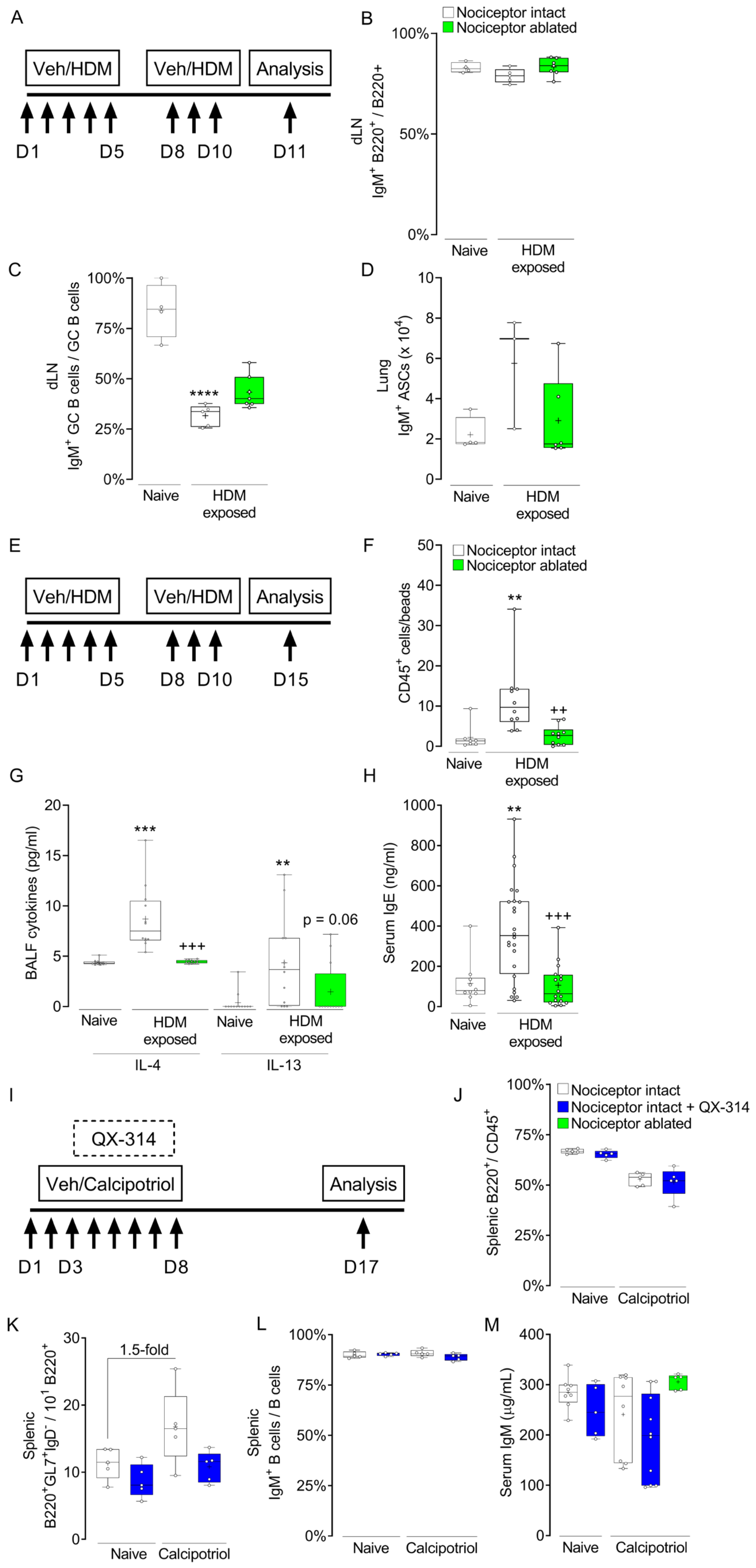
A
Veh/HDM
Veh/HDM
Analysis
D1
D5
D8
D10
D11
B
Nociceptor intact
Nociceptor ablated
dLN
IgM+ B220+ / B220+
100%
50%
0%
Naive
HDM exposed
C
dLN
IgM+ GC B cells / GC B cells
****
D
Lung
IgM+ ASCs (x 10^4)
E
D15
F
CD45+ cells/beads
**
++
G
BALF cytokines (pg/ml)
***
+++
**
p = 0.06
IL-4
IL-13
H
Serum IgE (ng/ml)
I
QX-314
Veh/Calcipotriol
D3
D17
J
Nociceptor intact + QX-314
Splenic B220+/ CD45+
Calcipotriol
K
Splenic B220+GL7+IgD- / 10^1 B220+
1.5-fold
L
Splenic IgM+ B cells / B cells
M
Serum IgM (µg/mL)

Supplemental Figure 4. Nociceptor ablation does not affect IgM and IgG1. (**A-D**) Allergic airway inflammation was induced by sensitizing WT and genetically ablated (TRPV1$^{Cre/wt}$::DTA$^{fl/wt}$) mice to intranasal HDM (20µg) as shown. Animals were sacrificed on day 11. Nociceptor ablation did not significantly affect (**B**) IgM^{+} total B cells or (**C**) IgM^{+} germinal center (GC) B cells in the draining lymph nodes (dLN) or (**D**) IgM^{+} antibody secreting cells (ASCs) in the lungs.

(**E-H**) Allergic airway inflammation was induced by sensitizing wildtype (WT) and genetically ablated (TRPV1$^{Cre/wt}$::DTA$^{fl/wt}$) mice to intranasal HDM (25µg) as shown. Compared to vehicle-exposed mice, HDM-challenge enhanced bronchoalveolar lavage fluid (BALF) numbers of (**F**) CD45^{+} cells, (**G**) type-2 cytokines as well as (**H**) serum levels of IgE. (**F-H**) These effects were reduced by the genetic ablation (TRPV1$^{Cre/wt}$::DTA$^{fl/wt}$) of airway nociceptor neurons).

(**I-M**) Calcipotriol or vehicle (EtOH) was applied to wildtype (WT) or genetically ablated (TRPV1$^{Cre/wt}$::DTA$^{fl/wt}$) mice. Groups of WT mice were additionally treated with QX-314 (10mg/mL, topical) as shown. Calcipotriol treatment did not increase the frequency of (**J**) B220^{+} B cell but raised the one of (**K**) splenic GL7^{+}IgD^{-} germinal center (GC) B cell by 1.5-fold. (**K**) Nociceptor silencing prevent raises in GL7^{+}IgD^{-} germinal center (GC) B cell. Calcipotriol treatment and nociceptor silencing or ablation did not significantly change (**L**) total splenic IgM^{+} B cells or (**M**) serum IgM levels.

*Graphs show range, median and "+" as mean. P-values determined using one-way ANOVA and Tukey's multiple comparisons test. * denotes comparison to vehicle exposed nociceptor intact mice and $^{+}$ to HDM-exposed nociceptor intact mice. P<0.05 is indicated by * or +; p<0.01 is indicated by ** or ++; p<0.001 indicated by +++; p<0.0001 indicated by ****. Representative experiments are shown; n=3-5/group. Experiments were replicated 1-3 independent times.*

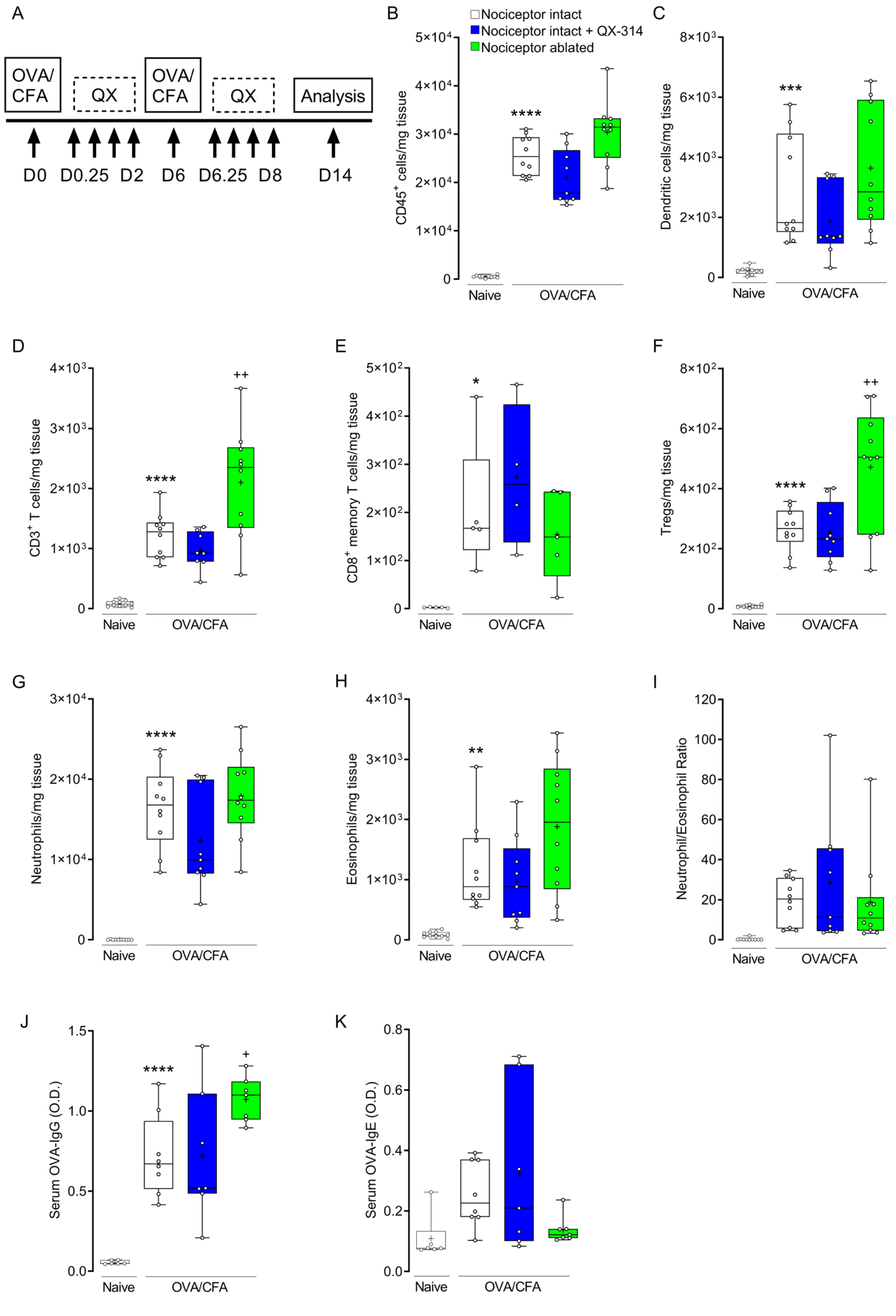
A
OVA/ CFA
QX
OVA/ CFA
QX
Analysis
D0
D0.25
D2
D6
D6.25
D8
D14
B
Nociceptor intact
Nociceptor intact + QX-314
Nociceptor ablated
CD45+ cells/mg tissue
Naive
OVA/CFA
C
Dendritic cells/mg tissue
D
CD3+ T cells/mg tissue
E
CD8+ memory T cells/mg tissue
F
Tregs/mg tissue
G
Neutrophils/mg tissue
H
Eosinophils/mg tissue
I
Neutrophil/Eosinophil Ratio
J
Serum OVA-IgG (O.D.)
K
Serum OVA-IgE (O.D.)

Supplemental Figure 5. Ablation or silencing of nociceptor neurons does not impact T_H1 skin immunity. (**A**) WT and genetically ablated (TRPV1$^{Cre/wt}$::DTA$^{fl/wt}$) mice were given subcutaneous (s.c.) sensitization with OVA (1mg/mL) in a 200µl emulsion of sterile PBS and 50% Complete Freud's Adjuvant (CFA) on day 0 and with 50% Incomplete Freud's Adjuvant on day 6. Groups of WT mice were additionally given QX-314 (10mg/mL, s.c.) at the site of injection every 12hrs for 48hrs after CFA/IFA sensitization. Animals were sacrificed on day 14.

In comparison to vehicle, OVA/CFA/IFA increased skin numbers of (**B**) CD45$^+$ cells, (**C**) dendritic cells, (**D**) CD3$^+$ T cells, (**E**) CD8$^+$ T cells, (**F**) regulatory T cells, (**G**) neutrophils, and (**H**) eosinophils. OVA/CFA/IFA immunization did not significantly change (**I**) neutrophil: eosinophil ratio or (**J**) serum OVA-IgE but increased (**K**) serum OVA-IgG. Sensory neuron ablation (TRPV1$^{Cre/wt}$::DTA$^{fl/wt}$) increased skin levels of CD3$^+$ and regulatory T cells and serum IgG levels, while nociceptor silencing (QX-314) had no impact on all tested parameters.

*Graphs show range, median and "+" as mean. P-values determined using one-way ANOVA and Tukey's multiple comparisons test. * denotes comparison to vehicle exposed nociceptor intact mice and $^+$ to HDM-exposed nociceptor intact mice. $P<0.05$ is indicated by * or +; $p<0.01$ is indicated by ** or ++; $p<0.001$ indicated by ***, $p<0.0001$ indicated by ****. N=5/group. Experiments were replicated 1-2 independent times.*

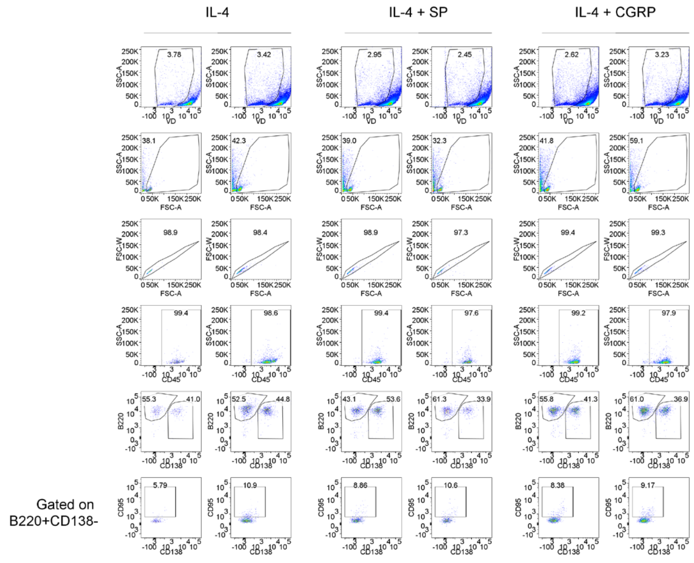

Supplemental Figure 6. Immunophenotyping B cells exposed to Substance P. Splenic B cells were isolated from naïve C57BL6 and cultured with or without LPS (10 μg/mL) and IL-4 (20 ng/mL) and exposed to vehicle, Substance P (1μM) and CGRP (300nM). After 4 days, the cells were immunophenotyped by flow cytometry and representative gating is shown.

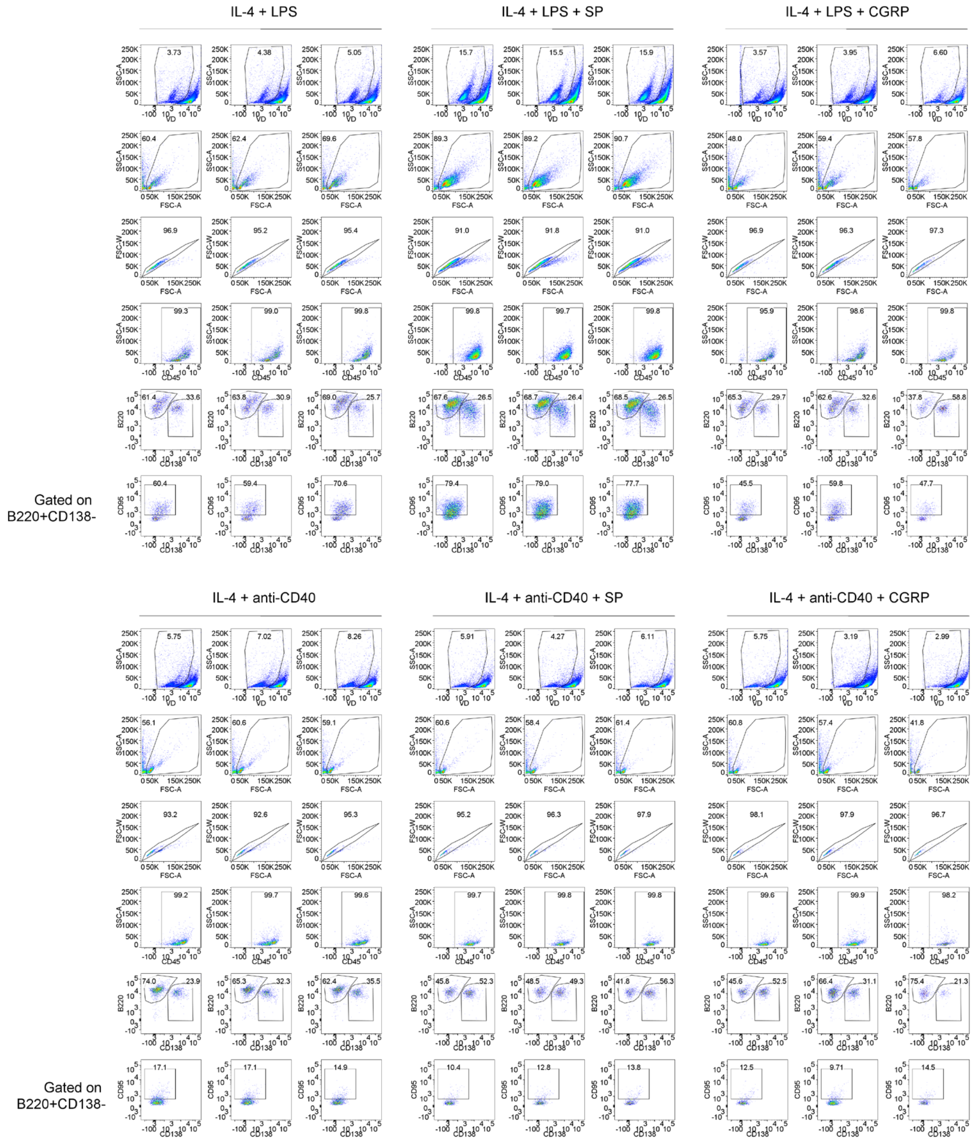

Supplemental Methods

Neuron Culture. Sensory neuron ablated (TRPV1$^{Cre/wt}$::DTA$^{fl/wt}$) or littermate control mice were sacrificed and their dorsal root ganglions (DRGs) were dissected and collected in DMEM medium (Corning 10-013-CV), completed with 50 U/mL penicillin and 50 µg/mL streptomycin (Fisher MT-3001-Cl). DRGs were incubated in HEPES buffered saline (Sigma, MO, USA) completed with 1 mg/mL collagenase IV (Sigma, # C0130) + 2.4 U/mL dispase II (Sigma 04942078001) for 80 minutes at 37°C and then triturated with glass Pasteur pipettes of decreasing size in supplemented DMEM medium, followed by centrifugation over a 10% BSA gradient in PBS and plating on Laminin (Sigma L2020) coated cell culture dishes. Neurons were cultured with Neurobasal-A medium (Gibco 21103-049) completed with 0.05 ng/µl NGF (Life Technologies 13257-019), 0.002 ng/µL GDNF (Peprotech 450-51-10), 0.01 mM AraC (Sigma C6645) and 200nM L-Glutamin (VWR 02-0131).

Calcium Imaging. Cultured neurons were used for calcium imaging 12-48 hours post-plating. Culture neurons were loaded with 10 µM Fura-2-AM (Life Technologies) at RT for 30-45 min in Neurobasal-A medium then washed by Standard Extracellular Solution (SES, 145 mM NaCl, 5 mM KCl, 2 mM $CaCl_2$, 1 mM $MgCl_2$, 10 mM glucose, 10 mM HEPES, pH 7.5), and imaged at RT. Ligands (1µM capsaicin (Sigma M2028; Tocris 0462), or 40 mM KCl (Sigma P3911) were applied for 10-30s directly onto neurons using perfusion barrels followed by buffer washout for 150 sec. Cultured neurons were illuminated by a UV light source (Xenon lamp, 75 watts, Nikon, NY, USA), 340 nm and 380 nm excitation alternated by an LEP MAC 5000 filter wheel (Spectra services, NY, USA), and fluorescence emission captured by Cool SNAP ES camera (Princeton Instruments, NJ, USA). 340/380 ratiometric images were processed, background corrected and analyzed with NIS-Elements (Nikon Instruments Inc.). Microsoft Excel and SigmaPlot (Systat Software, Inc) was used for further analyses.

Single Cell RNA Sequencing. Using the publicly available Broad Institute single-cell portal, we performed an *in-silico* analysis of single-cell RNA-sequencing of mouse lung CD45$^+$ immune cells (GEO access file: GSE127465). Genes queried were *Trpv1, Scn9a (Na$_V$1.8), Ptcra* and *CD3ε*. Individual cell data are shown as a $\log_2$ of 1 + (transcript per million/10). Experimental details and cell clustering were defined by Zilionis et al. (2019).

OVA/CFA Model. Mice were given subcutaneous (s.c.) sensitization with OVA (1mg/mL) in a 200µl emulsion of sterile PBS and 50% Complete Freud's Adjuvant (CFA) on day 0 and with 50% Incomplete Freud's Adjuvant on day 6, as previously described Foster et al. (2017). For mice in the QX-314 group, s.c. 10mg/mL QX-314 (Millipore Sigma: 112965-21-6) was given locally at site of injection every 12hrs for 48hrs after CFA/IFA sensitization. Mice were euthanized on day 14 by inhalation of carbon dioxide and blood was drawn from the heart for ELISA. Punch biopsy of the injection site skin was taken for flow cytometry.

Supplemental References

Foster, S.L., C.R. Seehus, C.J. Woolf, and S. Talbot. 2017. Sense and Immunity: Context-Dependent Neuro-Immune Interplay. *Front Immunol* 8:1463-1463.

Zilionis, R., C. Engblom, C. Pfirschke, V. Savova, D. Zemmour, H.D. Saatcioglu, I. Krishnan, G. Maroni, C.V. Meyerovitz, C.M. Kerwin, S. Choi, W.G. Richards, A. De Rienzo, D.G. Tenen, R. Bueno, E. Levantini, M.J. Pittet, and A.M. Klein. 2019. Single-Cell Transcriptomics of Human and Mouse Lung Cancers Reveals Conserved Myeloid Populations across Individuals and Species. *Immunity* 50:1317-1334.e1310.
